# Interventions for behaviour change and self-management in stroke secondary prevention: protocol for an overview of reviews.

**DOI:** 10.1186/s13643-018-0888-1

**Published:** 2018-12-13

**Authors:** Olive Lennon, Catherine Blake, Jo Booth, Alex Pollock, Maggie Lawrence

**Affiliations:** 1 School of Public Health, Physiotherapy and Sports Science, University College Dublin, Health Sciences Centre, Belfield, Dublin 4, Ireland; 2 School of Health and Life Sciences, Glasgow Caledonian University, Glasgow, UK; 3 Nursing, Midwifery and Allied Health Professionals Research Unit, Glasgow Caledonian University, Glasgow, UK

**Keywords:** Overview, Stroke, Secondary prevention, Behaviour change, Lifestyle, Self-management

## Abstract

**Background:**

Stroke secondary prevention guidelines recommend medication prescription and adherence, active education and behavioural counselling regarding lifestyle risk factors. To impact on recurrent vascular events, positive behaviour/s must be adopted and sustained as a lifestyle choice, requiring theoretically informed behaviour change and self-management interventions. A growing number of systematic reviews have addressed complex interventions in stroke secondary prevention. Differing terminology, inclusion criteria and overlap of studies between reviews makes the mechanism/s that affect positive change difficult to identify or replicate clinically. Adopting a two-phase approach, this overview will firstly comprehensively summarise systematic reviews in this area and secondly identify and synthesise primary studies in these reviews which provide person-centred, theoretically informed interventions for stroke secondary prevention.

**Methods:**

An overview of reviews will be conducted using a systematic search strategy across the Cochrane Database of Systematic Reviews, PubMed and Epistomonikas. Inclusion criteria: systematic reviews where the population comprises individuals post-stroke or TIA and where data relating to person-centred risk reduction are synthesised for evidence of efficacy when compared to standard care or no intervention. Primary outcomes of interest include mortality, recurrent stroke and other cardiovascular events. In phase 1, two reviewers will independently (1) assess the eligibility of identified reviews for inclusion; (2) rate the quality of included reviews using the ROBIS tool; (3) identify unique primary studies and overlap between reviews; (4) summarise the published evidence supporting person-centred behavioural change and self-management interventions in stroke secondary prevention and (5) identify evidence gaps in this field. In phase 2, two independent reviewers will (1) examine person-centred, primary studies in each review using the Template for Intervention Description and Replication (TIDieR checklist), itemising, where present, theoretical frameworks underpinning interventions; (2) group studies employing theoretically informed interventions by the intervention delivered and by the outcomes reported (3) apply GRADE quality of evidence for each intervention by outcome/s identified from theoretically informed primary studies. Disagreement between reviewers at each process stage will be discussed and a third reviewer consulted.

**Discussion:**

This overview will comprehensively bring together the best available evidence supporting person-centred, stroke secondary prevention strategies in an accessible format, identifying current knowledge gaps.

**Electronic supplementary material:**

The online version of this article (10.1186/s13643-018-0888-1) contains supplementary material, which is available to authorized users.

## Background

Annually, approximately 15 million people worldwide have a stroke [1] with global projections that the number of stroke survivors will rise to 77 million by 2030 [2]. Following transient ischaemic attack (TIA) or stroke, cardiovascular event rates are high: at 5 years, the risk of recurrent stroke is 18.3% and the risk of a cardiac event 6.8% and at 10 years following stroke the cumulative risk of recurrence is 39.2% [3], with higher death and disability noted with recurrent events [4]. Such high rates of cardiovascular morbidity and associated disability indicate the need for effective secondary prevention measures that address the population attributable risk. Modifiable risk factors have been identified in the INTERSTROKE study as hypertension, physical activity, dyslipidaemia, diet, central adiposity, psychosocial factors (stress (home and work), life events and depression), current smoking, cardiac causes, high or heavy episodic alcohol consumption and diabetes mellitus [5].

International best practice guidelines recommend multimodal approaches to secondary prevention that address medication prescription in conjunction with active provision of information and education regarding stroke, lifestyle risk factors, and medication adherence [6, 7]. To enhance effectiveness and compliance, it is recommended that these interventions are informed by behaviour change theory and incorporate behaviour change techniques [8]. Three main reasons are cited for advocating the use of theory-based interventions [9]. Firstly, interventions are more likely to be effective if they target causal determinants of behaviour and behaviour change. Secondly, theory can only be tested and further developed by evaluation of theoretically informed interventions. Thirdly, theory-based interventions facilitate an understanding of what works and thus are a basis for developing better theory across different contexts, populations and behaviours [9]. Furthermore, employment of explicit rather than implicit theoretical models provides a more consistent and generalisable framework within which to gather evidence that can drive implementation [10].

In long-term conditions such as stroke, self-management strategies are also critical. Defined as ‘the active management by individuals of their treatment, symptoms, lifestyle and the physical and psychological consequences inherent in living with a chronic condition’ [11], they have been shown to reduce morbidity and healthcare utilisation [10–13]. In stroke survivorship specifically, holistic self-management support interventions have aimed to empower individuals with the skills to (1) manage their medical condition; (2) maintain or change behaviours or life roles and (3) deal with the emotional consequences of survival [14]. It was noted previously that core components of self-management including goal setting, action planning and problem solving, when delivered as part of rehabilitation soon after stroke, affected a positive change in activities of daily living and a reduction in dependence/death [14]. However, a systematic review of outcome measures employed in self-management interventions in stroke survivorship identified that only one of the 13 studies included in the review employed an outcome measure that addressed secondary prevention [15], indicating under-utilisation of self-management strategies in secondary prevention stroke research.

Furthermore, the principles of a person-centred care (PCC) approach, now widely adopted in healthcare delivery [16], suggest that people should not be classified or treated according to their disease alone. Rather their subjective experiences in relation to their environment, situation and future plans need to be considered [17]. Tailoring interventions to individual (and family) needs and priorities has been identified in the literature as a key element of PCC design in stroke care [18]. Aggregated qualitative data examining stroke survivors’ perspectives of multimodal stroke secondary prevention interventions suggest three important PCC themes that warrant consideration: feeling supported, acquiring knowledge and gaining confidence [19].

**Fig. 1:**
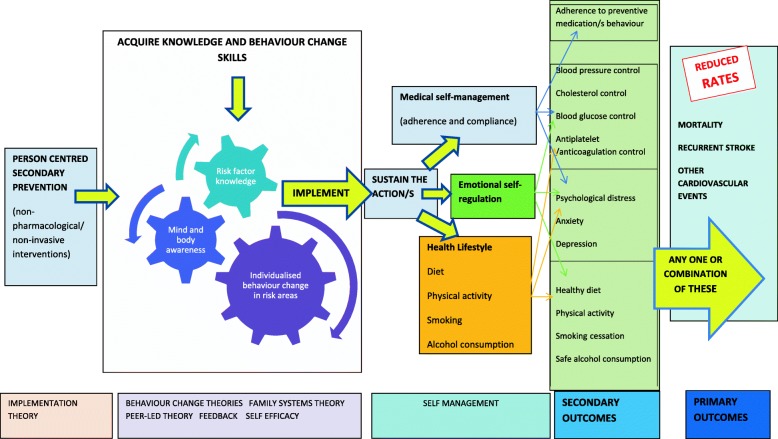
Model for person-centred, secondary stroke prevention behavioural change and self-management

It can then be argued that for optimal impact on mortality and morbidity rates in stroke secondary prevention, the prevention message must have contextual meaning to the individual and their wider support. The behavioural/lifestyle change/s required must then not only be adopted by the individual but sustained in the longer-term as a lifestyle choice, indicating the need for self-management interventions that draw on psychological theories of behaviour change to deliver education and skills training. To this end, we now propose a model, adapted with permission from Parke et al. (2015) [14] for effective person-centred secondary prevention following stroke that incorporates both theoretically informed behaviour change (risk modification) and self-management (Fig. 1). This model will be used as the basis on which to re-examine evidence relating to stroke secondary prevention which has previously been synthesised within published systematic reviews.

In the last 5 years, a growing number of systematic reviews in secondary prevention following stroke have addressed behavioural interventions [20–25] and self-management strategies [26]. Inherent methodological difficulties in systematic review of complex interventions are well documented in the literature [27–30] and include variations in the definition of behavioural change interventions and the theory underpinning these interventions. Identified reviews in stroke often employed different but overlapping terminology, e.g. ‘lifestyle interventions’ and ‘non-pharmacological interventions’ or ‘reduction of cardiovascular events secondary to stroke’ and ‘stroke secondary prevention’, with associated search and eligibility criteria reflecting these. Some reviews implemented criteria defining exercise as a mandatory component of the intervention/s delivered (modelled on the cardiac rehabilitation paradigm) [20, 23]. Other identified reviews included only studies in which the interventions focussed on educational or behavioural components targeting patients, healthcare providers and/or changes in the organisation of healthcare services excluding exercise [24], while others included varying combinations of all these components [21, 25]. From the perspective of the consumer of systematic reviews (e.g. healthcare practitioners, policy-makers), the lack of consistent terminology, methodological and theoretical differences, and inclusion of overlapping studies between these reviews makes identification of the mechanism/s that affect positive change difficult to identify or replicate clinically and can be overwhelming. To guide future research and clinical practice, there is a need to systematically and comprehensively bring together current evidence in a structured and consistent way. Therefore, an overview of reviews is proposed.

### Objectives

The primary aim of this synthesis is to provide a comprehensive overview of the evidence, mapping person-centred studies in included systematic reviews to the model proposed for secondary stroke prevention self-management (Fig. 1). This will enable us to address the following objectives:

- Summarise the available evidence by systematic review for behavioural and/or self-management interventions on outcomes of mortality, cardiovascular morbidity and modifiable risk factors after stroke.
- Identify unique primary studies addressing person-centred secondary prevention behavioural and self-management strategies, and highlight where published systematic reviews overlap by including the same primary studies
- Provide a detailed compendium of theoretically informed, replicable interventions employed in the primary studies identified. Intervention components reported in person-centred primary studies will be extracted under the item domains of the Template for Intervention Description and Replication (TIDieR) checklist including the theory underpinning the intervention [31].
- Determine the quantity and quality of evidence relating to theoretically informed studies employing behavioural and/or self-management strategies on mortality, cardiovascular end points, and secondary outcomes of interest, i.e. those identified in the secondary prevention self-management model (Fig. 1), by synthesising results of identified primary studies and assigning a GRADE of evidence for each outcome [32]
- Identify knowledge gaps and make recommendations for future research [33, 34]

## Methods

### Design

A systematic review of published reviews (referred to as an overview) will be conducted, informed by Cochrane guidelines for Overviews of Reviews [35]. This type of review has been identified previously as involving the systematic identification and retrieval of eligible reviews, assessment of bias (at review level) and integration of results from multiple systematic reviews [36].

**Fig. 2:**
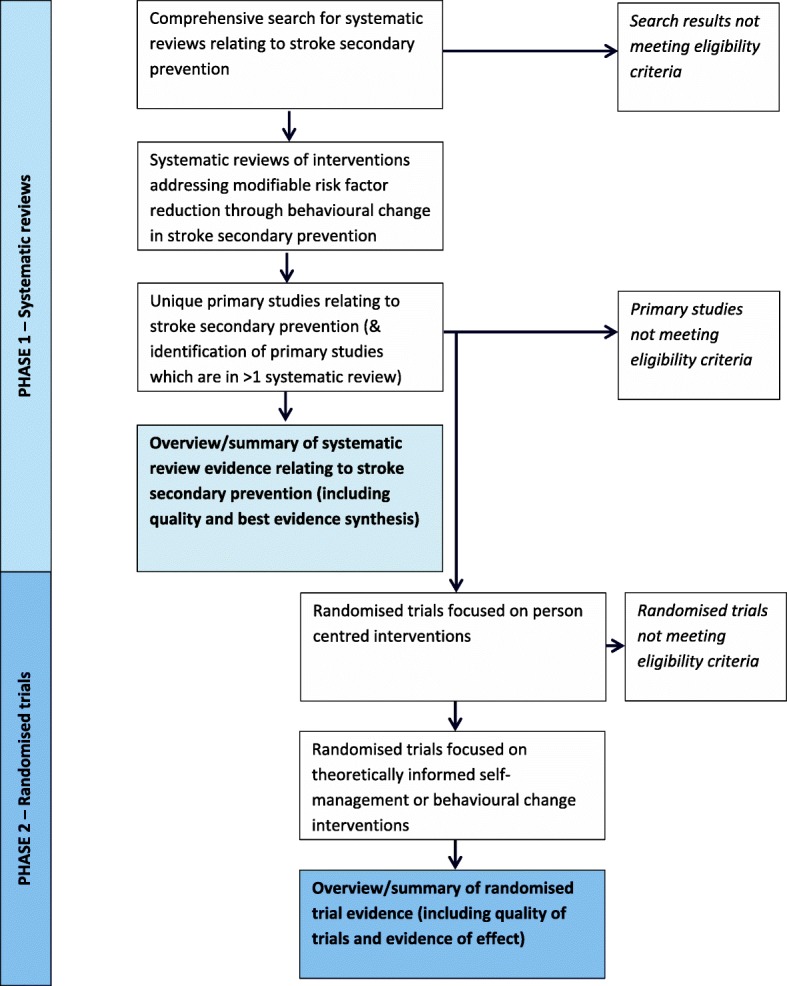
Outline of the two phases of the overview of reviews process

This overview will involve two phases, covering (1) identification, selection and appraisal of eligible systematic reviews; and (2) identification, selection, appraisal and synthesis of eligible primary studies from within the systematic reviews (see below for eligibility criteria). Figure 2 details the flow process for the methodology employed.

### Search methods

A comprehensive (broad) *search strategy* using subject headings and keywords will be used to search key databases, e.g. Cochrane Library of Systematic Reviews, PubMed and Epistomonikos to identify potentially eligible systematic reviews (Additional file 1). Comprehensive search strings, using controlled vocabulary and free-text terms, addressing stroke or TIA and secondary prevention/risk factor management will be developed. Search syntax and Boolean operators (AND; OR) will be used, as appropriate. For databases not specific to systematic reviews, we will add a third methodological search string. A sample search strategy is included as Additional file 1. Peer review of the electronic search strategy will be conducted in line with best practice PRESS guidelines [37].

In addition to the electronic search strategy, reference lists of identified reviews will be hand searched, as will reference lists of relevant guideline and policy documents [6, 7, 38].

In phases 1 and 2 described in the methodology sections below, each process will be conducted independently by two reviewers. Where disagreement between reviewers is noted there will be discussion between these reviewers. A third reviewer will be consulted where no consensus has been reached through discussion.

### Phase 1—Systematic reviews

#### Selection criteria

Systematic reviews that meet the following criteria will be considered:

- The population of interest comprises individuals post stroke or TIA, or cardiovascular disease populations where stroke is a discrete sub-population
- Some or all of the data synthesised has been extracted from person-centred interventions aimed at risk factor reduction following stroke
- The included studies were randomised controlled trials (RCTs) and/or cluster randomised controlled trials (CRCTs). If reviews included other study designs, we will include those from which we can extract RCTs /CRCTs data only
- Included intervention/s aimed at changing health behaviours after stroke, at an individual or population level. Change behaviours may include any one or combination of the following: secondary prevention medication adherence (anti-hypertensive agents, cholesterol lowering agents, antiplatelet/anticoagulant agents); healthy diet; physical activity participation; absolute smoking cessation; safe alcohol consumption; and psychosocial stress management
- Included intervention/s reported the effect of behavioural change on mortality, recurrent stroke or other cardiovascular events, or—in the absence of these outcomes—on health behaviours or modifiable physiological risk factors as a precursor to risk reduction (as above)

Reviews will be excluded if they included only studies that aim to change the behaviours of health professionals in secondary prevention practice or health systems interventions which aim to improve secondary prevention protocols.

### Identification of reviews

For papers identified by the search strategy, screening for inclusion (title, abstract and full-text stages) will be conducted independently by two reviewers (OL and ML).

### Data extraction from included reviews

Data will be extracted independently by two reviewers using an agreed proforma which will include the following items:

- Protocol related: Bibliographic data; publication dates covered by the review; stated aims and objectives; databases employed in the review; key interventions considered and the definition/s that described these interventions; inclusion and exclusion criteria applied.
- Results related: number of primary studies included in the review; outcomes reported at review level, relevant to this overview (see below); meta-analyses by intervention and outcome at review level relevant to this overview (see below).
- Alignment and overlap between reviews: the objective of each systematic review and primary studies in each review will be tabulated. Unique primary studies will be identified, and any primary studies which are included in more than one review will be ascertained.
- Outcomes: The outcomes of interest presented below are summarised in the secondary prevention self-management model presented in Fig. 1.

Primary outcomes of interest are: Reduction in mortality (all cause and cardiovascular), recurrent stroke and other cardiovascular events post intervention and at time points of 6 months, 1 year, 5 years and 10 years after the intervention delivery.

Secondary outcomes include sustained health behaviours post intervention and at time points of 6 months, 1 year, 5 years and 10 years post intervention in one or more of the following: medication adherence, healthy diet, physical activity participation, smoking cessation, safe alcohol consumption and psychosocial stress management.

Tertiary outcomes include physiological measurements of sustained and optimal blood pressure control, cholesterol control, blood glucose control and anticoagulation control post intervention and at time points of 6 months, 1 year, 5 years and 10 years after intervention delivery.

## Results

Meta-analysis results, where data were pooled from interventions delivered to the individual post stroke and relating to outcomes of interest to this overview, will be extracted from included reviews and the Grading of Recommendations Assessment, Development and Evaluation (GRADE) criteria [32] applied (Table 1).

**Table 1:** Grading of Recommendations Assessment, Development and Evaluation

Grade recommendation	Interpretation
High quality	Where further research is unlikely to change confidence in the estimate of effect
Moderate quality	Where further research is likely to impact on confidence in the estimate of effect
Low quality	Where further research is very likely to have an important impact on confidence in the effect size and is likely to change the estimate
Very low quality	Where great uncertainty about the estimate exists

### Quality appraisal of included reviews

Each review will be independently rated for quality by two reviewers using the risk of bias in systematic reviews ROBIS tool [39]. As the primary authors (OL and ML) have both published reviews in this area, the quality assessment will be conducted by independent third parties.

### Best evidence synthesis of reported systematic review results

A best evidence synthesis relating to meta-analyses conducted in primary and secondary outcomes of interest to this overview will be compiled. Where two or more reviews report the same outcome for the same intervention, the best evidence decision will be by consensus of authors and based on the risk of bias identified during quality appraisal, the degree of overlapping studies and the timeframe in which the review was conducted. All decisions reached through this process will be systematically documented and transparently reported. Knowledge gaps relating to stroke secondary prevention using person-centred behavioural change/self-management approaches will be identified in this process.

### Phase 2—Primary studies

#### Identification and screening of primary studies

Unique primary studies identified from included reviews during phase 1 will be screened independently by two reviewers to ensure they meet the following specific study-level inclusion criteria:

- RCT or CRCT study design
- Stroke or TIA population
- Person-centred intervention delivery only
- Include primary, secondary or tertiary outcomes related to this overview

Studies which include interventions designed to alter care delivery processes or health professional education in tandem with a person-centred delivery will be excluded at this stage.

#### Identification of replicable, theoretically informed interventions

Following initial screening, the intervention described in included primary studies (and associated protocol paper, where relevant), will be independently examined and extracted using domains 1 and 2 of the 10 items comprising TIDieR’s checklist. These first two domains comprise the rationale and theoretical framework underpinning the intervention [31].

### Data extraction from primary studies

Identified primary studies that employ theoretically informed self-management and/or behaviour change interventions will have the following data items extracted independently by two reviewers.

- Characteristics of the participants (e.g. number; age, gender, stroke aetiology, stroke severity and time post stroke)
- Additional characteristics relating to the intervention (using domains 3–10 of the TIDieR checklist)
- Primary, secondary and tertiary outcomes (that are of interest in this overview)
- Results of between group comparisons

### Quality appraisal of primary studies

The two reviewers will assess the quality of each primary study using the Cochrane Risk of Bias Tool.

### Data synthesis

Data from primary studies which employ a theoretical framework of behaviour change and/or self-management will be grouped by intervention type and by outcomes of mortality, cardiovascular endpoints and secondary outcomes of interest identified in Fig. 1. Where data type and outcomes from two or more primary studies permit, a meta-analysis will be conducted. For continuous data change scores with standard deviation of the difference or standard mean differences will be calculated from pooled data. For dichotomous variables risk ratios with 95% confidence intervals using the Mantel-Haenszel method will be employed. The quality of the evidence for each intervention type and outcome group identified across studies will be reported based on the Grading of Recommendations Assessment, Development and Evaluation (GRADE) criteria [32]. Judgement of GRADE quality for each comparison will be agreed by consensus of all authors, with consideration of risk of bias of included studies [40], inconsistency [41], indirectness [42], imprecision [43] and publication bias [44].

### Registration

This manuscript acts as the public record of the current review of reviews. Therefore, to avoid duplication, the protocol has not been registered with PROSPERO.

## Discussion

While international guidelines recognise the need to implement non-pharmacological risk reduction strategies post stroke, the mechanism/s to achieve sustained lifestyle changes and the impact of these changes on mortality and cardiovascular endpoints remains unclear. It is recognised that systematically reviewing complex interventions is challenging [27–30]. Published reviews in secondary stroke prevention have reflected this, where variations across the reviews in the interventions considered and grouped together and/or the grouping of multiple theoretical approaches make it difficult for healthcare practitioners and policy-makers to identify the components of interventions that are successful and affect positive change or replicate these clinically. It is hoped that this overview of reviews will allow the quantity and quality of the evidence for theoretically informed interventions in behaviour change and/or self-management behaviours in stroke secondary prevention to now be summarised by intervention type and outcome.

One methodological difficulty identified in the literature in synthesising findings in an overview of reviews is overlap, where the same primary studies appear in more than one systematic review [45]. A number of solutions have been proposed, the simplest of which, as identified by McKenzie and Brennan (2017) [36], is to include only one systematic review (or meta-analysis) addressing each question. But identified reviews in secondary stroke prevention rarely address identical questions and selecting one review from multiple could result in loss of important data or entire studies. An alternative solution, which is to report results from multiple reviews addressing the same or similar questions, and which overlap at the individual study level, brings added complexity where re-analysis is required [36]. In phase 1 of this overview, we aim to synthesise review results by reporting the best available evidence by outcome using results from only one included review. In phase 2, however, we adopt a more novel approach to address this issue, namely, to drill down to primary study level in each review, consider whether the study is eligible to the overview question and the model for person-centred stroke secondary prevention self-management, conduct our own meta-analysis by intervention type and outcome, thus eliminating overlap without loss of studies.

Overviews of reviews as a methodological approach are a relatively new and developing field. Controversy remains as to whether an overview has the capacity to identify evidence gaps in an area. Ballard (2017) [45], for example, in a recently published scoping review of methodological guidance for overviews, concluded that an overview cannot fulfil this function. Findings from five exemplar reviews however have clearly concluded that overviews had successfully identified gaps in the evidence [34]. The authors of this overview, by identifying the current available evidence by systematic review that supports the published best practice recommendations in non-pharmacological stroke secondary prevention, aim to identify where knowledge gaps in theoretically informed lifestyle and self-management interventions in secondary stroke prevention arise and hope to make recommendations for future research based on these findings. The addition of a second phase in the review process, where GRADE criteria for data drawn from primary studies matching stricter criteria with respect to their rationale and theoretical basis, will allow a novel comparison and contrast of two different methodological approaches to data synthesis in an overview of reviews.

## Additional file

Additional file 1: Sample PubMed search strategy. (DOCX 17 kb)

## Abbreviations

GRADE: Grading of Recommendations Assessment Development and Evaluation
RCT: Randomised controlled trial
ROBIS: Risk of bias in systematic review
