# Supplementary material for: Interventions for behaviour change and self-management in stroke secondary prevention: protocol for an overview of reviews
Source: Syst Rev. 2018 Dec 13;7:231. doi: 10.1186/s13643-018-0888-1 (PMC6292177; doi:10.1186/s13643-018-0888-1)
Supplement: Supplementary file 1 — Sample PubMed search strategy. (DOCX 17 kb) [file 13643_2018_888_MOESM1_ESM.docx]

**Sample Pubmed Search Strategy**

**Stroke**

1. cerebrovascular disorders/ or exp basal ganglia cerebrovascular disease/ or exp brain ischemia/ or exp carotid artery diseases/ or exp intracranial arterial diseases/ or exp intracranial arteriovenous malformations/ or exp "intracranial embolism and thrombosis"/ or exp intracranial hemorrhages/ or stroke/ or exp brain infarction/ or vasospasm, intracranial/ or vertebral artery dissection/

2. (stroke or poststroke or post-stroke or cerebrovasc$ or brain vasc$ or cerebral vasc$ or cva$ or apoplex$ or SAH).tw.

3. ((brain$ or cerebr$ or cerebell$ or intracran$ or intracerebral) adj5 (isch?emi$ or infarct$ or thrombo$ or emboli$ or occlus$)).tw.

4. ((brain$ or cerebr$ or cerebell$ or intracerebral or intracranial or subarachnoid) adj5 (haemorrhage$ or hemorrhage$ or haematoma$ or hematoma$ or bleed$)).tw.

5. 1 or 2 or 3 or 4

**Risk reduction**

6. health education.mp. or exp Health Education/

7. health promotion.mp. or exp Health Promotion/

8. health behavior.mp. or exp Health Behavior/

9. secondary prevention.mp. or exp Secondary Prevention/

10. counseling.mp. or exp Counseling/

11. counsel$.mp.

12. (health adj5 (educat$ or program$ or promotion$ or behavio?r)).tw.

13. (patient adj5 (educat$ or program$)).tw.

14. 6 or 9 or 11 or 8 or 7 or 10 or 12 or 13

15. ((secondary or multifactor$) adj3 (prevention or intervention)).tw.

16. (risk adj3 factor$ adj5 (reduc$ or manag$ or intervent$)).tw.

17. (lifestyle adj3 (intervent$ or advice)).tw.

18. (life?style adj3 (intervention$ or advice or alter$ or educat$ or chang$)).tw.

19. (behavio?r$ adj3 chang$).tw.

20. (health?care adj3 advice).tw.

21. non?pharmacologic$.tw.

22. 15 or 16 or 17 or 18 or 19 or 21 or 20

23. 14 or 22

24. 5 and 23

25. ethanol.mp. or exp Ethanol/

26. (alcohol$ or ethanol$ or wine or beer or spirit$ or ((problem or hazardous or harmful) adj3 drink$)).tw.

27. 26 or 25

28. exp Tobacco/ or "Tobacco Use Cessation"/ or tobacco.mp.

29. exp smoking/

30. exp smoking cessation/

31. (tobacco or smok$).tw.

32. 31 or 29 or 30 or 28

33. diet$.tw.

34. (healthy adj3 eating).tw.

35. (diet adj3 chang$).tw.

36. 34 or 33 or 35

37. exercise.mp. or exp Exercise/

38. (physical adj3 activ$).tw.

39. 37 or 38

40. 27 or 32 or 36 or 39

41. 40 and 24

42. limit 41 to humans

**Systematic reviews**

43. meta-analysis/

44. literature review/

45. systematic review.pt.

46. meta?analy$.tw.

47. ((systematic or quantitative or methodolog$) adj (overview$ or review$)).tw.

48. integrative research review$.tw.

49. 43 or 44 or 45 or 46 or 47 or 48

50. 49 and 42
